# *Flavobacterium branchiophilum* and *F. succinicans* associated with bacterial gill disease in rainbow trout *Oncorhynchus mykiss* (Walbaum) in water recirculation aquaculture systems

**DOI:** 10.1111/jfd.12249

**Published:** 2014-04-10

**Authors:** C Good, J Davidson, G D Wiens, T J Welch, S Summerfelt

**Affiliations:** 1 The Conservation Fund's Freshwater InstituteShepherdstown, WV, USA; 2 National Center for Cool and Cold Water AquacultureKearneysville, WV, USA

**Keywords:** bacterial gill disease, flavobacteria, rainbow trout, recirculation aquaculture

**Figure 1:**
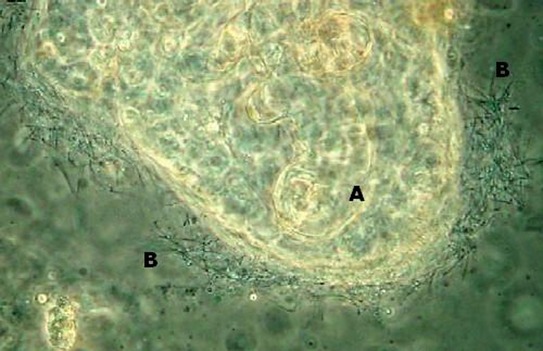
Typical appearance of bacterial gill disease, as observed through light microscopy (wet-mount, 400× magnification, phase-contrast), with gill tissue (A) (in this field, the distal secondary lamella) covered with numerous, clustered, hair-like bacteria (B) morphologically resembling *Flavobacterium branchiophilum*.

Bacterial gill disease (BGD) is a common and occasionally devastating disease that affects numerous cultured fish species throughout the world (Starliper & Schill 2012). Outbreaks of BGD tend to occur when environmental conditions deteriorate, and opportunistic pathogens can more readily cause overt disease (Bullock 1972; Schachte 1983). The putative causative agent, *Flavobacterium branchiophilum*, has been shown to induce BGD under laboratory conditions (e.g. Lumsden *et al*. 1995; Ostland *et al*. 1995); however, diagnosis of BGD in the field is generally carried out through light microscopy and/or observation of clinical signs. Therefore, the identity of the bacterial specie(s) present on the gills of BGD-affected fish in culture settings is most often based on visual assessment alone. The observed bacteria using microscopy are presumed to be *F. branchiophilum* based on organism morphology (numerous long, thin rods; Fig. 1.), with this presumption supported by previous BGD research (e.g. Byrne *et al*. 1995; Ostland *et al*. 1997; Derksen, Ostland & Ferguson 1998) and various *F. branchiophilum*-specific diagnostic approaches that have been developed. The latter include ELISA (MacPhee *et al*. 1995), IFAT (Heo, Kasai & Wakabayashi 1990), and PCR (Toyama, Kita & Wakabayashi 1996). A comprehensive examination of bacterial species present on the gills of naturally infected fish, however, has not been carried out and is desirable to enhance our understanding of BGD and to inform further research (e.g. potential vaccine development). We therefore sought to induce BGD through environmental manipulation (as opposed to pathogen challenge) and to identify the bacterial species involved in typical BGD outbreaks. Our findings are presented in this short communication.

We raised rainbow trout *Oncorhynchus mykiss* (Walbaum) in six replicated water recirculation aquaculture systems (WRAS) as well as three smaller circular flow-through tanks. The WRAS were all operated at ‘near-zero’ exchange (i.e. 76-day system hydraulic retention time), with three systems receiving water ozonation and three without ozonation; for an in-depth description of the WRAS utilized, please refer to Davidson *et al*. (2011). The trout were raised to 175 days post-hatch (105 ± 3.62 g mean weight ± standard error). To promote BGD, environmental conditions in all six WRAS were compromised via (i) disruption of the circular tank water rotational current to reduce solids removal through the bottom center drain, (ii) doubling feeding rates to promote accumulation of waste feed and further water quality deterioration and (iii) reduction of tank dissolved oxygen (through reduced liquid oxygen flow through the low-head oxygenators) to approximately 70% saturation. Water temperature ranged from 16 to 18 °C during the study period. Within a period of 1 month, mild BGD outbreaks (i.e. relatively low mortality) occurred in all three non-ozonated WRAS; no BGD or suspicious mortality was observed in the ozonated WRAS. For each episode of BGD, gill tissue sampling was carried out on the following: (i) six BGD-affected fish (confirmed visually with wet-mount light microscopy of right-side gill tissue, with BGD presenting typically as large numbers of bacteria resembling *F. branchiophilum*); and (ii) from the same WRAS experiencing BGD, six fish not displaying BGD signs and no bacteria resembling *F. branchiophilum* observed on the gill via light microscopy. At the same time as (i) and (ii) above, six fish (non-BGD, as assessed above) were also randomly selected from (iii) the nearest ozonated WRAS and (iv) a randomly selected flow-through tank. For all gill sampling, fish were first humanely killed with 200 mg L^−1^ tricaine methanesulfonate (MS-222; Western Chemical Inc.), and uniform segments of the second gill arch (left side) were then carefully removed with sterile instruments and placed in 2-mL cryogenic vials. The vials were then snap-frozen in liquid nitrogen and stored at −80 °C for subsequent laboratory analyses.

All frozen gill samples were shipped overnight on dry ice to the Core for Applied Genomics and Ecology (CAGE), University of Nebraska (Lincoln, NE). Briefly, each gill sample was washed for 30 min in 0.1% peptone, and the bacteria were harvested by centrifugation. Bacterial DNA was then extracted using the standard CAGE protocol (i.e. bead-beating, followed by use of Qiagen Stool Kits), and DNA from each sample was used for PCR amplification of the V1–V3 regions of 16S rRNA genes using the F8 (Felske *et al*. 1997) and R518 (Muyzer, Waal & Uitterlinden 2003) universal primers. Primers for each sample contained the A/B titanium sequencing adapters and a barcode unique to each sample. Purified amplicons from 96 samples were pooled and subjected to pyrosequencing using the Roche 454 GS-FLX Titanium chemistry. Extracted DNA and subsequent amplicons were measured for concentration prior to PCR and sequencing, respectively, to aim for PCR band saturation; amplicons were pooled equally such that sequencing depth (i.e. number of reads per sample) was nearly equal for all samples. Raw sequence data were then divided into respective sample (bar-code) bins. The resultant FASTA files of the binned data were further processed by CAGE through two pipelines: (i) CLASSIFIER pipeline, which produced a single spreadsheet with counts for each taxonomic rank for each sample, and (ii) operational taxonomic unit (OTU) pipeline yielding individual spreadsheets for each OTU cut-off from 97% (Species), 95% (Genus), 90% (family/class) and 80% (phylum).

**Figure 2:**
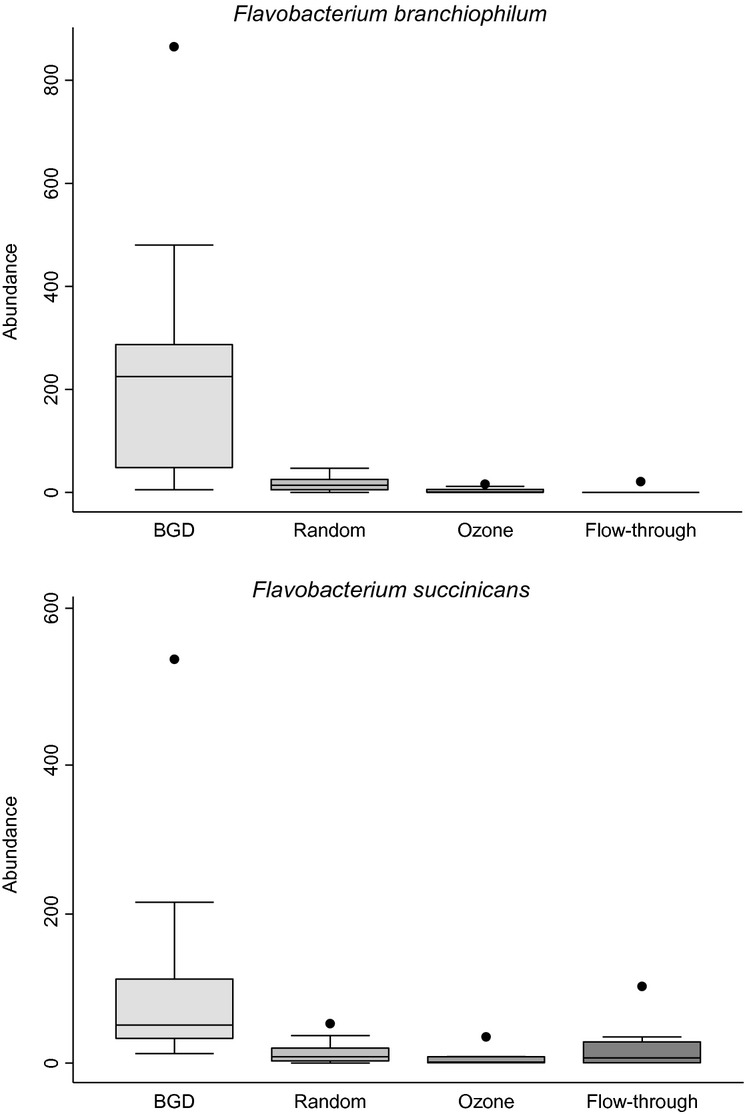
Box-and-whisker plots illustrating abundance counts of *Flavobacterium branchiophilum* and *F. succinicans* on rainbow trout gills within each of the four groups sampled. Abundance counts represent number of sequences within each bacterial species-level operational taxonomic unit (OTU). The horizontal line within each box represents the median abundance; the top and bottom of each box represent the upper and lower quartiles, respectively; whiskers above and below each box represent 1.5× the interquartile range; and dots represent outliers.

The initial intent was to determine the identities and abundance of all bacterial species on the gill samples; however, when reviewing the data, it was determined that bacterial contamination had occurred during sample processing, resulting in contamination with DNA from various lactic acid bacteria including *Lactobacillus*, *Weissella* and *Leuconostoc*. Further investigation identified the source of contamination as the 0.1% peptone buffer for removing bacteria from the gill samples. Despite this set-back, no bacteria of the genus *Flavobacterium* were identified in the contaminated peptone, and therefore, all *Flavobacterium* sequences were selected and binned from the 454 sequencing reads by CLASSIFIER. Following removal of sequences <150 or >1000 bases in length, the sequencing reads were split into species-level OTUs with CD-HIT-EST at a cut-off score of 0.97, as is commonly used to define species-level relatedness at the molecular level using 16S rRNA. Overall, there were 75 species-level OTUs of the *Flavobacterium* genus, with a total of 6305 reads among all samples. Of the 75 *Flavobacterium* OTUs, 5253 reads (83.3%) were associated with two OTUs; best hit-to-species, using BLASTn against the NCBI non-redundant nucleotide database, revealed that these two most prevalent species were *F. branchiophilum* and *F. succinicans*. The relative abundance of each bacterial species within each experimental group was obtained from the raw sequence counts within each species-level OTU. Both species were significantly associated (*P* < 0.05 by ANOVA) with BGD-affected fish (Table 1; Fig. 2); additionally, *F. branchiophilum* was significantly more prevalent in random non-affected fish sampled from BGD tanks than those sampled from ozonated WRAS and flow-through tanks.

**Table 1:** Mean (± standard error) abundance counts for *Flavobacterium branchiophilum* and *F. succinicans* on the gills of bacterial gill disease (BGD)-affected rainbow trout, plus fish randomly sampled from BGD-affected tanks (Random), ozonated systems (Ozone) and non-water recirculation comparison tanks (flow-through), following 454 pyrosequencing of sampled gill tissues. Abundance counts represent mean (± standard error) number of sequence counts within each bacterial species-level operational taxonomic unit (OTU). Different superscripts within each bacterial species represent significant (*P* < 0.05) differences

	Abundance			
	BGD	Random	Ozone	Flow-through
*F. branchiophilum*	248 ± 65.2^a^	16.6 ± 4.10^b^	3.50 ± 2.32^c^	2.63 ± 2.63^c^
*F. succinicans*	106 ± 39.5^a^	14.3 ± 4.73^b^	6.88 ± 4.23^b^	21.8 ± 12.5^b^

These results confirm that *F. branchiophilum* is the dominant bacterial species present on the gills of rainbow trout affected by natural, environmentally induced BGD. The other major fish pathogens of the genus Flavobacterium that are known to colonize gills, namely *F. columnare* and *F. psychrophilum*, were not among the dominant OTUs associated with disease and identified to the species-level, despite their occasional previous detection at the research facility. The detection of *F. succinicans* at significantly higher levels in BGD-affected fish, in conjunction with significantly higher levels of *F. branchiophilum*, is also novel and supports the assertion by Bernardet and Bowman (2006) that *F. succinicans* may be a commensal species with the potential to act as an opportunistic pathogen under certain conditions (e.g. BGD). Fish subclinically infected with *F. branchiophilum* (i.e. the ‘random’ trout not displaying overt disease signs, sampled from BGD-affected tanks) possessed significantly higher levels of *F. branchiophilum* on their gills, compared with the ozone and flow-through groups, but relatively ‘normal’ levels of *F. succinicans*, which further suggests that *F. branchiophilum* is the primary BGD pathogen, with *F. succinicans* proliferating once overt disease is established. Very little has been published concerning the association of *F. succinicans* with disease in fish; Anderson and Ordal (1961) isolated *F. succinicans* from the external lesions of salmon and their holding water, and in a study of wild, feral and farmed fish of Michigan, USA, *F. succinicans* and related strains were frequently recovered from both coldwater and warmwater fishes, predominantly from gill tissue (Loch *et al*. 2013). Additional research is necessary to elucidate the relationship between *F. branchiophilum* and *F. succinicans* and their respective roles in the transition from healthy to diseased fish under suboptimal environmental conditions. Indeed, recent research by Zamora *et al*. (2012, 2013) and Loch *et al*. (2013) suggests that a plethora of *Flavobacterium* species may be associated, as opportunistic pathogens, with disease in farmed fish, and therefore, further research is necessary to complete our understanding of the ecology of the *Flavobacterium* genus in relation to disease in cultured fishes. The recent report of the genome sequence of *F. branchiophilum* strain FL-15 will facilitate the association of identified virulence factors with disease severity (Touchon *et al*. 2011).

Further research is required to confirm the association of *F. branchiophilum* and *F. succinicans* with BGD at other aquaculture sites, particularly during non-instigated outbreaks with high mortality, and among other species naturally affected by this disease. In pursuit of the original goal of this study, a more complete examination (i.e. deep 16S rRNA sequencing) of all bacterial species, beyond those of the *Flavobacterium* genus, on the gills of fish affected by naturally occurring BGD, is also required to increase our understanding of this disease. This knowledge, in turn, can be used to develop novel remedial or preventative strategies to combat BGD as a major source of mortality in the aquaculture industry.
